# The Experiences of Informal Caregivers of People With Dementia in Web-Based Psychoeducation Programs: Systematic Review and Metasynthesis

**DOI:** 10.2196/47152

**Published:** 2023-05-29

**Authors:** Ying Yu, Lily Xiao, Shahid Ullah, Claudia Meyer, Jing Wang, Anne Margriet Pot, Fathimath Shifaza

**Affiliations:** 1 College of Nursing and Health Sciences Flinders University South Australia Australia; 2 College of Medicine and Public Health Flinders University Bedford Park South Australia Australia; 3 Bolton Clarke Research Institute Melbourne, Victoria Australia; 4 Ageing and Independent Living Research Centre Monash University Melbourne, Victoria Australia; 5 Centre for Health Communication and Participation La Trobe University Melbourne, Victoria Australia; 6 Faculty of Nursing, Health Science Center Xi’an Jiao tong University Xi’an, Shanxi China; 7 Erasmus School of Health Policy & Management Erasmus University Amsterdam Amsterdam Netherlands; 8 Optentia North-West University Vanderbijlpark South Africa

**Keywords:** informal caregivers, dementia, psychoeducation, online, web based, qualitative research, systematic review, metasynthesis

## Abstract

**Background:**

Informal caregivers of people living with dementia experience a higher level of physical and mental stress compared with other types of caregivers. Psychoeducation programs are viewed as beneficial for building caregivers’ knowledge and skills and for decreasing caregiver stress.

**Objective:**

This review aimed to synthesize the experiences and perceptions of informal caregivers of people with dementia when participating in web-based psychoeducation programs and the factors that enable and impede informal caregivers’ engagement in web-based psychoeducation programs.

**Methods:**

This review followed the Joanna Briggs Institute protocol of systematic review and meta-aggregation of qualitative studies. We searched 4 English databases, 4 Chinese databases, and 1 Arabic database in July 2021.

**Results:**

A total of 9 studies written in English were included in this review. From these studies, 87 findings were extracted and grouped into 20 categories. These categories were further synthesized into 5 findings: web-based learning as an empowering experience, peer support, satisfactory and unsatisfactory program content, satisfactory and unsatisfactory technical design, and challenges encountered in web-based learning.

**Conclusions:**

High-quality and carefully designed web-based psychoeducation programs offered positive experiences for informal caregivers of people living with dementia. To meet broader caregiver education and support needs, program developers should consider information quality and relevancy, the support offered, individual needs, flexibility in delivery, and connectedness between peers and program facilitators.

## Introduction

Dementia is a major cause of disability among older adults worldwide [1]. People living with dementia have complex care needs and are often highly dependent on others to care for them [2]. Most people living with dementia are cared for by unpaid informal caregivers who are their family members or friends. Worldwide, in 2019, informal caregivers spent approximately 5 hours per day per person with dementia assisting in daily living activities [2]. They experienced a higher level of physical and mental stress compared with other types of caregivers; showed increased caregiver burden, anxiety, and depression; and showed decreased quality of life [3-5]. Such caregiving situations directly impact the caregiver’s ability to provide quality care.

Early educational interventions to prepare informal caregivers for their caregiver role are crucial [2,6]. However, the educational interventions offered do not always meet their needs. Informal caregivers often feel that they lack knowledge of dementia progression and symptom management and the skills for providing daily care [7,8]. They also expressed the need for more support at home from trained health professionals [9] that could foster knowledge sharing; build skills, such as symptom management and physical care; and provide emotional support [10,11].

Psychoeducation programs are viewed as beneficial for meeting caregivers’ expectations and learning needs through knowledge and skill building, while encouraging positive thoughts, decreasing caregiver stress, and improving caregivers’ psychological well-being and quality of life [12]. According to Cheng et al [12], psychoeducation programs usually incorporate theoretical, psychological, and behavioral training components relevant to dementia care to achieve these benefits. Traditionally, psychoeducation programs are delivered face-to-face in small groups [12]. Web-based psychoeducation programs have been widely used in recent years to offer convenience and flexibility to increase caregivers’ participation and retention [13-15]. However, many informal caregivers reported a lack of time or flexibility to commit to these programs because of care responsibilities [16].

Despite the known advantages of web-based psychoeducation for caregivers, underutilization and a lack of program trustworthiness have been identified [2]. Furthermore, studies have revealed a high dropout rate among caregivers in web-based psychoeducation programs [17]. The reasons for the high dropout rate varied across studies and programs. For example, the low recruitment and retention rates reported in a study by Baruah et al [18] indicated a need for further adaptations to the program to improve acceptability and accessibility. Whereas, other studies have indicated that gender [19], program length [20], and uncertain factors [21] contributed to the dropout rate. There is a need to synthesize studies on caregivers’ experiences of using web-based psychoeducation programs to gain further insights into their experiences and facilitators affecting participation in a global context. This review addresses this gap in the literature.

This review aims to synthesize (1) the experiences and perceptions of informal caregivers of people with dementia when participating in web-based psychoeducation programs and (2) the factors that enable and impede informal caregivers’ engagement in web-based psychoeducation programs.

## Methods

### Inclusion and Exclusion Criteria

This review included studies that reported components of the experiences of informal caregivers of people living with dementia when using web-based psychoeducation programs in a home care setting. The review included qualitative studies and mixed methods studies that included qualitative components. The following studies were excluded from the review: (1) quantitative design; (2) web-based programs without an educational component, such as social support groups (ie, singing group) and telehealth; (3) non–internet-based programs, such as a DVD or booklet; (4) the population of interest in the study was people with dementia in residential care or hospital settings, rather than home care settings; and (5) not written in English, Chinese, or Arabic (because of team members’ backgrounds).

### Search Strategy and Screening Method

Keywords were identified according to the study’s population (informal caregivers of people living with dementia), interest (web-based psychoeducation program), and context (home care setting; Multimedia Appendix 1). A Boolean search was conducted by combining keywords. The following English databases were searched in July 2021: CINAHL, Web of Science, MEDLINE, and Scopus (Multimedia Appendix 2). Keywords were translated into Chinese (by YY) and Arabic (by FS) by the review team. The Chinese databases searched included the China National Knowledge Infrastructure, Wang Fang Data, Weipu Data, and Chaoxing Data. We also manually searched the *Academic Journal of the Middle East* for articles written in Arabic. No time limit was applied to the search. All retrieved records were imported into EndNote 20 [22] and Covidence [23] to remove duplicate studies. In total, 4 reviewers (YY, LX, CM, and SU) screened the English titles and abstracts. In addition, 2 reviewers (YY and JW) screened the Chinese titles and abstracts to identify studies that met the inclusion criteria, and 2 reviewers (YY and LX) reviewed the full text retrieved. The reference list of each selected article was scanned manually.

### Assessment of Methodology

The methodology of all selected papers was assessed using Joanna Briggs Institute (JBI) critical appraisal instruments for qualitative research [24]. The review team decided to include only those studies that satisfied >5 appraisal questions. The main findings from each paper were critiqued by 2 reviewers to evaluate the level of credibility (ranked as unequivocal, credible, or not supported) according to JBI [24]. The final synthesized findings were derived from unequivocal (findings and supporting data are beyond reasonable doubt and therefore not open to challenge) and credible (findings and supporting data lack clear association and are therefore open to challenge) findings. Throughout the quality assessment process, disagreements between any 2 reviewers were resolved either through comparison and discussion between the reviewers or through a third reviewer.

### Data Extraction

Qualitative data were extracted by 2 reviewers (anonymized for peer review) using the standardized data extraction tool from JBI Qualitative Assessment and Review Instrument [24]. The tool includes (1) author, publication year, and country; (2) participants’ characteristics and sample size; (3) web-based education or training program details, including duration, facilitator details, and theoretical framework; (4) study setting, design, and methods; and (5) main findings. The main findings from each study were extracted with an illustration to evaluate the credibility of the findings (Multimedia Appendix 3) [25-33].

### Data Synthesis and Reporting

Data synthesis in this review followed the JBI protocol of meta-aggregation of qualitative studies [24], with the following three steps: (1) the main findings from each study were reviewed by 2 reviewers to evaluate the level of credibility, with unequivocal and credible findings included in the data synthesis and meta-aggregation; (2) similar findings were grouped into categories; and (3) categories were refined and synthesized into final findings. The final findings were reported following PRISMA (Preferred Reporting Items for Systematic Reviews and Meta-Analyses) 2020 [34] (Multimedia Appendix 4).

## Results

### Study Inclusion

**Figure 1:**
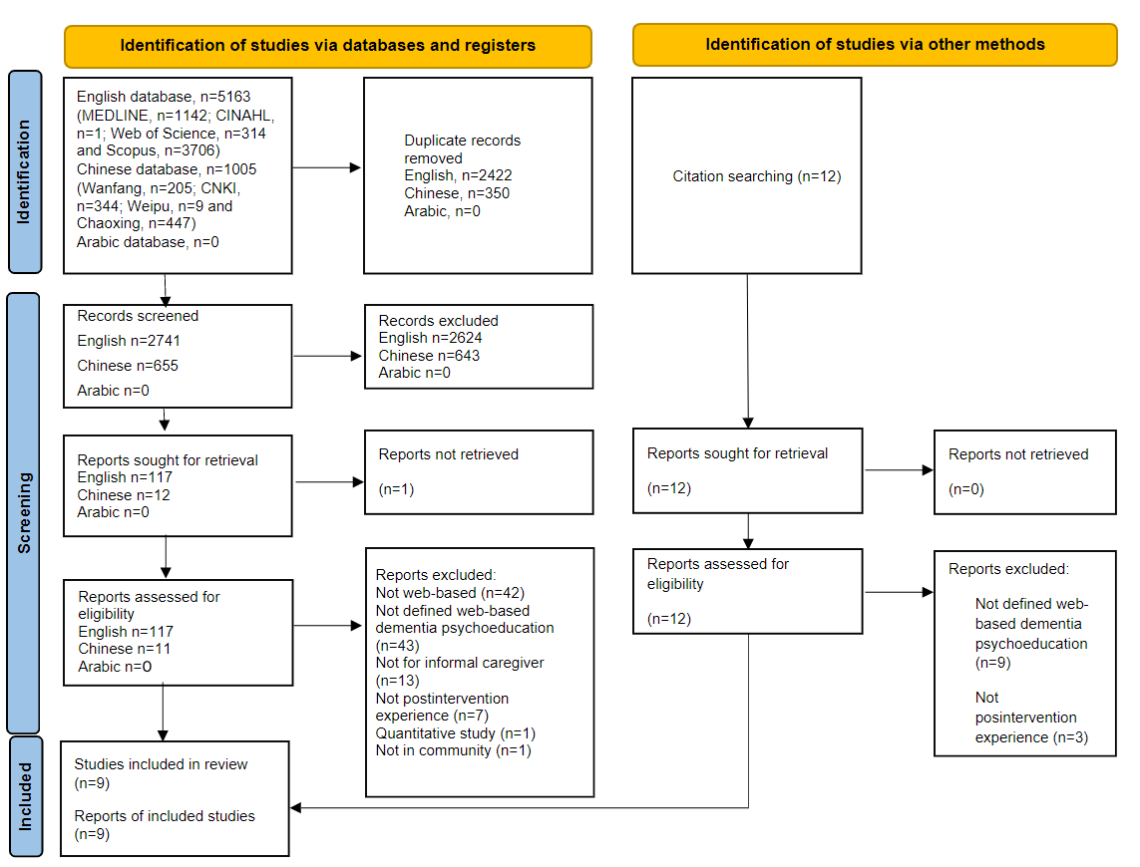
PRISMA (Preferred Reporting Items for Systematic Reviews and Meta-Analyses) flow diagram. CNKI: China National Knowledge Infrastructure.

A total of 6168 articles were initially identified from database searches and uploaded to Covidence [23] (English databases, n=5163; Chinese databases, n=1005; and Arabic database, n=0). Covidence automatically removed duplicates (n=2422). Duplicates were manually removed from the Chinese database (n=350). After a title and abstract screening (English, n=2721; Chinese, n=655; and Arabic, n=0), 128 (English, n=117 and Chinese, n=11) full-text articles were retrieved. An additional 12 articles were identified from the searching the reference list of the included articles. After assessing the eligibility of full-text articles, 9 studies written in English met the inclusion criteria and were included for methodology assessment. No articles written in Chinese or Arabic met the inclusion criteria. The study selection process is illustrated in Figure 1.

### Methodology Quality

The methodological quality of the 9 selected studies was assessed and is presented in Table 1. Of the 9 studies reviewed, 5 (56%) were mixed methods studies and 4 (44%) were qualitative studies. Only 1 study indicated the philosophical perspectives underpinning the methodology [25]. In total, 3 studies were not clear about the cultural and theoretical orientations of the researcher [26-28], and 5 studies did not address the researchers’ influence on the study [26,27,29-31]. Moreover, 1 study only selected 2 cases to represent the qualitative data collected [30]. Therefore, the adequate representation of the participants in that study is questionable. All studies, except 1, indicated participation in an ethics review [27]. No studies were excluded from this review based on the methodological quality assessment.

**Table 1:** Quality appraisal for qualitative studies.

Study	Q1^a,b^	Q2^c^	Q3^d^	Q4^e^	Q5^f^	Q6^g^	Q7^h^	Q8^i^	Q9^j^	Q10^k^
Brennan et al [29]	U^l^	Y^m^	Y	Y	Y	Y	U	Y	Y	Y
Duggleby et al [33]	U	Y	Y	Y	Y	Y	Y	Y	Y	Y
Fowler et al [30]	U	Y	Y	Y	Y	Y	U	U	Y	U
Gaugler et al [26]	U	Y	Y	Y	Y	U	U	Y	Y	Y
Halbach et al [27]	U	Y	Y	U	Y	U	U	Y	U	Y
Hattink et al [28]	U	Y	Y	Y	Y	U	Y	Y	Y	Y
Kovaleva et al [31]	U	Y	Y	Y	Y	Y	U	Y	Y	Y
Lewis et al [32]	Y	Y	Y	Y	Y	Y	Y	Y	Y	Y
Ploeg et al [25]	Y	Y	Y	Y	Y	Y	Y	Y	Y	Y

^a^Q: question.

^b^Q1: Is there congruity between the stated philosophical perspective and the research methodology?

^c^Q2: Is there congruity between the research methodology and the research question or objectives?

^d^Q3: Is there congruity between the research methodology and the methods used to collect data?

^e^Q4: Is there congruity between the research methodology and the representation and analysis of data?

^f^Q5: Is there congruity between the research methodology and the interpretation of results?

^g^Q6: Is there a statement locating the researcher culturally or theoretically?

^h^Q7: Is the influence of the researcher on the research, and vice versa, addressed?

^i^Q8: Are participants, and their voices, adequately represented?

^j^Q9: Is the research ethical according to the current criteria or, for recent studies, and is there evidence of ethics approval by an appropriate body?

^k^Q10: Do the conclusions drawn in the research report flow from the analysis, or interpretation, of the data?

^l^U: unclear.

^m^Y: yes.

### Characteristics of the Included Studies

The included studies were published between 1991 and 2019 and were conducted in the United States (n=5), Canada (n=2), the Netherlands (n=1), and Norway (n=1; Table 2). A total of 367 people participated in the qualitative component of these studies. Among the included studies, 5 used a mixed methods design and 4 applied a qualitative study design. The methodologies used in those studies included case studies [27,30], qualitative descriptions [25,31], content analysis of open-ended questions in the survey [26,29,31,32], and secondary analysis of telephone interviews [33]. The data collection methods used in these studies included focus group interviews [27], semistructured interviews either via telephone or face-to-face [25,30,31], or surveys with open-ended questions [26,29,31,32].

**Table 2:** Characteristics of the included studies.

Study, country	Study design	Program	Participants in the qualitative study	Method	Findings
Brennan et al [29], United States	Mixed methods	ComputerLink	Family caregivers of PwD^a^ (n=22)	Data collection: Messages posted on the discussion forum were collected Data analysis: Qualitative content analysis of collected messages	Pros: The forum and questions and answers section served as emotional support and social interaction opportunities for caregivers of PwD The public communication section allows participants to control the discussion focus and address the issue in a timely manner Self-paced learning provided more flexible learning for caregivers without time and space restraints Cons: Findings do not represent a diverse population (ie, different age groups and cultural backgrounds)
Duggleby et al [33], Canada	Secondary analysis of a mixed methods study	MT4C^b^	Family caregivers of PwD (n=92)	Data collection: Telephone interviews Data analysis: Qualitative content analysis	Pros: Improved PwD’s self-efficacy Cons: Poor internet connectivity and low computer literacy were the barriers to accessing MT4C Reasons for not using the program also included caregiver demands and preference for a paper or a face-to-face interaction
Fowler et al [30], United States	Case study	Virtual health care neighborhood technology	Family caregivers of PwD used the program (n=28)	Data collection: Interviews Data analysis: Qualitative descriptive	Pros: Provided social support and information sharing using the blog section. The blogs included safety, sleep issues, memory, social engagement, enjoyment, and suggestions. Participants have opportunities to interact with health professionals from different disciplines Cons: Only reported 2 cases
Gaugler et al [26], United States	Mixed methods	CARES Dementia Care for Families	Family caregivers of PwD (n=41)	Data collection: Survey Data analysis: Qualitative content analysis of open-ended questions in the survey	Pros: Comprehensive content Use of real individuals with dementia in videos The video shows the stages/progression of dementia The flexibility of web-based delivery Cons: The video segment was too small Some audio segment was incomplete
Halbach et al [27], Norway	Qualitative case report	Mobile app mYouTime	Relatives and staff of PwD (n=17)	Data collection: Focus group interviews Data analysis: Qualitative descriptive	Pros: Well-structured learning units Large font size Contains videos Contains basic and in-depth information Cons: The quiz section was the least attractive Need more local information rather than be redirected to another web page
Hattink et al [28], the Netherlands	Mixed methods	The Digital Alzheimer Center	Family caregivers of PwD (n=6), PwD (n=6), and professional staff (n=6)	Data collection: Observations Web-based survey Semistructured interviews Data analysis: Thematic analysis	Pros: Clear layout, calm background, large font, and contrasting color Comprehensive and well-written information Helped caregivers of PwD with understanding and dealing with dementia Information can be accessed anytime and anywhere (flexibility in delivery) Cons: Posting a message on the forum, finding information on driving, and watching videos appeared difficult to some participants Small sample size
Kovaleva et al [31], United States	Qualitative description	Tele-Savvy	Family caregivers of PwD (n=36)	Data collection: Interviews Data analysis: Qualitative content analysis	Pros: Provided opportunity for caregivers to connect with others via videoconferences (peer support and learning from others) The web-based program promoted access for those who need to travel Videoconferencing was facilitated by a health professional Contains prerecorded expert-delivered lessons Provided caregiver manual Cons: Information needs to be more relevant to stage-specific caregiving Need more videos to cover more complex situation and represents more diverse cultural backgrounds The program needs to be longer Videoconferences need to be longer Videoconferencing needs to be more engaging Technical issues (poor internet connection) Insufficient instructions on how to join videoconferences Need more detailed written and illustrated instructions for video viewing The web-based program may not be suitable for some people. The study only included people who have internet access
Lewis et al [32], United States	Mixed methods	The internet-based Savvy Caregiver program	Family caregivers of PwD (n=47)	Data collection: Survey with open-ended questions Data analysis: Constant comparative analysis	Pros: Information and caregiving strategies were relevant and interesting to participants Videoclips of professionals, caregivers, and PwD The convenience of the internet program Presentation of the program Cons: Spelling errors Technical difficulties (difficulty in navigating the website) Repetition of information Length of the program Did not provide an opportunity for participants to interact with other people Need a hard copy workbook
Ploeg et al [25], Canada	Qualitative	MT4C	Family caregivers of PwD (n=56)	Data collection: Semistructured, open-ended, and telephone interviews Data analysis: Qualitative content analysis	Pros: Easy to navigate Provided the opportunity to reflect on and share their caregiving experiences Information was relevant and applicable to the individual caregiver’s situation Provided affirmation of their caregiving experiences through the content of the website and linked videos Cons: MT4C did not apply to the caregiver’s current situation or suit their current needs because of their stage in the caregiving journey Technical issues and security concerns Writing or sharing their thoughts and experiences in MT4C Need a directory of services searchable by postal code Not having a person available to answer caregivers’ questions Not having a navigator to help the caregiver identify and access resources that meet their specific needs

^a^PwD: people with dementia.

^b^MT4C: My Tools 4 Care.

### Content of the Psychoeducation Programs

A total of 8 programs were reported in 9 studies (Table 3). In total, 2 studies reported the same program from different perspectives [25,33]. For example, Duggleby et al [33] only reported the reasons of nonusers in the web-based MT4C program, whereas Ploeg et al [25] reported users’ experiences in the same program. All programs were asynchronized web-based psychoeducation programs and had a clear indication of the educational component [25-33]. A total of 4 programs offered peer support functions [28-31]; of these 4 programs, 3 used asynchronized discussion forums or blogging [28-30] and 1 applied a synchronized method such as videoconferencing [31]. Health professionals’ involvement in the programs was reported in 4 studies [28-31]. Moreover, 1 study reported a program in a mobile app format, with limited details of psychoeducational content [27]. The duration of the psychoeducation programs ranged from 7 weeks [31], 2 months [28], 3 months [25,30,33] to 12 months [29]. Overall, 3 studies did not specify a time frame for participants to view or test the program [26,27,32].

**Table 3:** Details of the psychoeducation programs.

Study		Program and duration		Delivery format	Program content	
Brennan et al [29]		ComputerLink; 12 months		Asynchronized, web based	Content: dementia care information, decision support, and communication Theoretical framework: multiattribute utility theory Theoretical training: yes Psychological training: unclear Behavior training: unclear Peer support: using private email and discussion forum Facilitator: discussion forums were facilitated by health professionals.	
Duggleby et al [33] and Ploeg et al [25]		My Tool 4 Care; 3 months		Asynchronized, web based	Content: each web page contains frequently asked questions, resources, and a calendar. An electronic copy of the Alzheimer Society’s Alzheimer disease booklet was available. Theoretical framework: Meleis’ theory of transition Theoretical training: yes Psychological training: yes Behavior training: yes Peer support: not offered Facilitator: not offered	
Fowler et al [30]	Virtual Health Care Neighbourhood; 3 months		Asynchronized, web based			Content: information relevant to caring for people with dementia at home Theoretical framework: not indicated Theoretical training: yes Psychological training: yes Behavior training: yes Peer support: using Question and Answer and Social Support forums Facilitator: the blogging section was supported by health professionals.
Gaugler et al [26]		The CARES for Families; the duration was not indicated		Asynchronized, web based	Content: information on understanding memory loss, living with dementia, and using the CARES Approach Theoretical framework: not indicated Theoretical training: yes Psychological training: yes Behavior training: yes Peer support: not offered Facilitator: not offered	
Halbach et al [27]		mYouTime mobile app; the duration was not indicated		Asynchronized, web based	Content: lectures, videos, and hyperlinks about dementia care. Details were not discussed in the paper. Theoretical framework: not indicated Theoretical training: unclear Psychological training: unclear Behavior training: unclear Peer support: not offered Facilitator: not offered	
Hattink et al [28]		The Digital Alzheimer Center; the duration was not indicated		Asynchronized, web based	Content: information about dementia, an overview of appointments, community sections, news, and upcoming events Theoretical framework: not indicated Theoretical training: yes Psychological training: yes Behavior training: yes Peer support: using the forum Facilitator: participants can privately email health professionals or make an appointment.	
Kovaleva et al [31]		Tele-Savvy; 7 weeks		Hybrid, asynchronized, web-based information with synchronized videoconferencing for peer support	Content: prerecorded expert-delivered lessons about dementia care Theoretical framework: social cognitive theory and stress and coping theory Theoretical training: yes Psychological training: yes Behavior training: yes Peer support: weekly instructor-facilitated videoconferences Facilitator: health professionals	
Lewis et al [32]		Internet-based Savvy Caregiver program; the duration was not indicated		Asynchronized, web based	Content: information on (1) the effects of dementia on thinking, (2) taking charge and letting go, (3) providing practical help, and (4) managing daily care and difficult behavior Theoretical framework: stress and coping theory Theoretical training: yes Psychological training: yes Behavior training: yes Peer support: not offered Facilitator: not offered	

### Synthesized Findings

#### Overview

A total of 87 findings were extracted and grouped into 20 categories based on similarities and differences. These were further synthesized into five findings: (1) web-based learning as an empowering experience, (2) peer support, (3) satisfactory and unsatisfactory program content, (4) satisfactory and unsatisfactory technical design, and (5) challenges encountered in web-based learning (Multimedia Appendix 5). These synthesized findings are built on evidence rated as moderate to high confidence, which is outlined in the ConQual summary of findings in Multimedia Appendix 6. Multimedia Appendix 7 presents the meta-aggregation flowchart.

#### Synthesized Finding 1: Web-Based Learning as an Empowering Experience

This synthesized finding was based on 9 findings from 7 studies [26-32]. Caregivers who used web-based psychoeducation programs had a sense of empowerment through the knowledge they gained, and activities were undertaken [28-30]. For example, 1 participant stated the following [30]:

Being a part of the study at that time in my life really helped me cope with difficult family issues and decisions.

Caregivers welcomed topics on caregiver coping skills, which helped them develop strategies to deal with everyday challenges [29,32]. One participant stated the following [32]:

It is a gentle reference vehicle to understanding Alzheimer’s changes. It won’t smack you in the face with the fear of what is coming but will prepare you for techniques to cope.

Caregivers perceived that the knowledge they learned through real-life stories enabled them to understand the disease, which improved their self-efficacy [26-28]. One participant stated the following [26]:

The examples and the stories of families who live with Alzheimer’s were very informative and gave me comfort that I, too, can do this.

Some programs encouraged participants to complete their behavioral appraisal and develop a long-term plan [25]:

It [MT4C] made me even realize somebody else needs a list of doctors and [chuckles] you know, things like that... It made me think about personal care in the future because that’s long-term care.

Some caregivers were initially intimidated by web-based learning, but their experience in a well-run web-based classroom encouraged them to engage with the program [31]:

At first I was... this is not gonna work; I’m 60 years old. It really worked, I loved going to school online, I thought I was in a real class—I’m talking a real classroom.

Similarly, another participant indicated the following [30]:

I was a little intimidated with it at first but then I got on and it worked very smoothly, you know, the way it was supposed to and it made the experience kind of fun.

Participants expressed that having a program facilitator to answer their questions may further enhance their experience [25]. For example, 1 participant commented the following [25]:

Having a person available to answer caregivers’ questions by telephone and having a navigator to “be that bridge” to help the caregiver identify and access resources that meet their specific needs.

Overall, web-based psychoeducation programs empowered participants by enhancing their self-efficacy, skill building, knowledge sharing, and self-reflection, which contributed to a positive learning experience.

#### Synthesized Finding 2: Peer Support

This synthesized finding was based on 9 findings identified from 6 studies [25,28-32]. Peer interactions were important factors that influenced caregivers’ experiences in the web-based psychoeducation program. Asynchronized peer support included web-based forums for participants to exchange information and was perceived positively by participants [28-30]. One participant stated [29]:

There are frequent statements of encouragement and support among caregivers for example “My husband is in the middle stages of the disease and I would like some suggestions on how to occupy his time...” “Dorothy I also have a problem with my wife who likes to walk and gets bored...” “Hi this is Sue. I noticed a reply to idle Time,...”

Reading fellow caregivers’ stories provided an opportunity for caregivers to share, reflect on, and have a better understanding of dementia care. For example, 1 participant wrote the following [30]:

Oh, I’m not out here alone, kind of thing but just to be able to see what other people’s stories were like, how others were handling things and seeing how people interacted with each other. That medium was really valuable.

Encouragement and support from synchronized online peer support groups were also considered helpful [31]. However, not all peer support was positive. Issues identified in synchronized online peer support groups were more apparent. Poor group interactions were reported in 1 study that used videoconferences and negatively influenced caregivers’ experience. Group members were not focused on the topic, and a lack of equal opportunity to contribute to the group meeting and a desire to have more interactions were reported [31].

When the program did not offer a peer support function, participants specifically commented on the value of connecting and sharing experiences with others [25,32]. For example, 1 participant commented the following [32]:

I don’t have the option of sharing or interacting with others. The opportunity for questions related to my situation are not possible.

Caregivers also suggested the following [25]:

Adding a feature to MT4C to enable caregivers to connect with one another to share information, experiences, and caregiving strategies would be helpful.

Caregivers perceived that a facilitator played a crucial role in motivating them and clarifying the issues discussed in peer support groups [31]:

One of the very helpful parts of the chats was to have positive feedback from the teachers. I don’t think caregivers get very many “good job on that” ... comments. It is easy to know when we mess up ... hard to know that we did it well.

Peer support during the program reduced caregivers’ feelings of isolation, and many participants expressed a desire to stay connected after the program ended [31]:

For me it was a lifesaver... seeing all those people from all around the country... they are not really handling it any better than I ... I don’t feel so alone in spirit.

#### Synthesized Finding 3: Satisfactory and Unsatisfactory Program Content

This synthesized finding was based on a total of 17 findings identified from 7 studies [25-29,31,32]. The program content aspects considered in this finding include program components, such as video, and the information presented in the video or text format, such as different topics covering dementia caregiving strategies. No content was delivered in a synchronized format in the included studies.

A video component was welcomed by most participants, especially when a real person with dementia and their caregivers were featured in the video [32]. Videos enhanced caregivers’ understanding of dementia progression and care needs at different stages [26,27,31]. One caregiver stated [26]:

I really liked the videos that showed the progression of the disease in the early, middle, and late stages of the disease. For example, the making coffee and taking a bath example. I also liked the driving example, too, about the different parts of the brain and how they are affected.

Other caregivers echoed similar comments [32]:

Person with dementia was very interesting and I felt like I could connect with them.

The video structure and content also contributed to caregivers’ experiences. Although some programs’ videos were well structured [27], in other programs, the video display was too small [26], had poor audio quality [27], and content lacked cultural diversity [31]. Additional videos to highlight more challenging situations were requested by participants in 1 study [31]; for example:

The Caucasian daughter (age 61) suggested the vignettes did not portray the “messiness of life”—times when a care recipient may not follow caregiver’s guidance, multiple family members involved in caregiving, and families with limited resources: I would have liked to see a daughter or son single caregiver with just a parent, try to make it more identifiable and inclusive.

Caregivers perceived that the information provided in the web-based psychoeducation program was important. They welcomed information that accommodated their individual learning needs [27,28,31]. One caregiver stated [32]:

Good information, I found myself surprised at being able to relate to a lot of it.

Participants also perceived that the information provided should be relevant to the individual caregiver’s needs and their caregiving journeys [26] and detailed and practical [25]. One participant stated the following [25]:

I feel like I’m not there yet; Mom’s still early, so some of the things are a bit more advanced...

They particularly liked the information presented by both caregivers and experts [32]. The participants also noted that some programs missed important topics [25,27,31]. One participant stated [27]:

It was a known issue that the 23 lectures were not covering the entire area, and this was also remarked on with several participants mentioning missing topics and in-depth information

Most participants in this review were satisfied with the video content and written information included in web-based psychoeducation programs.

#### Synthesized Finding 4: Satisfactory and Unsatisfactory Technical Design

This synthesized finding came from a total of 23 findings identified from 6 studies [25-28,31,32]. The program design aspects considered in this finding include structure, language, functionality, accessibility, and supplementary material.

Participants liked a clear page layout with a large font size for the content [27,28,32]. A lack of systematic layout was reported in 1 study [31]:

Participants suggested that the [printed] manual be laid out more clearly (e.g., include a table of contents and a glossary) and be more precisely coordinated with the videos, videoconference “lectures,” and “homework” assignments.

Participants identified grammar and spelling errors in 2 programs [27,32]. There were also concerns about the literacy level of 1 program [25]:

It is a lot of text and the literacy level. Oh, the other thing is it’s only in English... you need to make the language a bit simpler.

One caregiver suggested that the case scenario presented needed to be positive to provide a better learning experience [26]:

I found it very sad to be left with the vision of the dear man peeling bananas. You could have chosen something a bit more uplifting.

Caregivers in 1 program considered quizzes to be the least helpful component [27]. Participants in another program experienced information overload and were frustrated by lengthy, repetitive, and missing content [32].

Caregivers especially welcomed the flexibility, convenience, and easy navigation of psychoeducation programs delivered on the web [28]:

You can check this information anytime, even in the middle of the night.

These features were extremely helpful for caregivers who lived far from the place where a face-to-face program might be delivered [31]:

I live forty miles from everywhere; it was wonderful... It was good to be able to do it online rather than trying to get in the car, considering the traffic situation here.

The caregivers expressed that the program website should have a bookmark function [32]. Supplementary materials, such as instruction manuals, were also suggested by the participants when they were not provided [31,32]. Caregivers would also like ongoing access to the program after completion for various reasons [26,30,31]. One caregiver stated [31]:

Caregivers could not access the videos after Tele-Savvy conclusion; however, many stated that they would be willing to rewatch videos, share them with family members, and rewatch them when their care recipient is in a later dementia stage.

#### Synthesized Finding 5: Challenges Encountered in Web-Based Learning

This synthesized finding was based on a total of 7 findings identified from 4 studies [25,28,31,33]. Technical issues such as problems with accessing and poor internet connection were a great challenge in using 2 web-based programs [31,33], which did not differentiate between asynchronized programs (information accessing) and synchronized online peer support. One participant commented the following [33]:

My internet connection at home is poor—I live in a rural area.

Others experienced problems during synchronized videoconferencing; or example [31]:

Problems during videoconferences (e.g., poor Internet connection, slow sound and video transmission, and insufficient instructions on joining videoconferences) affected connectedness.

A low level of computer literacy among the participants also contributed to access difficulties [31,33]. One participant commented the following [31]:

Some caregivers noted that others struggled to follow some directions... and needed to be better aligned relative to their webcam and sit in a position with good lighting.

Caregivers who struggled with the technology seem to prefer hard copy information [33]:

Sometimes, you actually have to have something printed in front of you, uh, and I’m better off—I’m better with paper.

Time was another challenge in this regard. Caregiving demands prevented some from participating in web-based psychoeducation programs [25,28,33]. One participant commented the following [33]:

[I] work full-time early morning to late evening... and at the end of the day, I don’t have the energy or time to go on the computer.

Similarly, another carer stated [25]:

The more time I spend on the computer, the more [name of spouse] approaches me and saying “What are you doing? Why aren’t you sitting with me?”

Other caregivers preferred learning through actual social contact [31]:

It would have been better to absorb the content in a group setting, person to person... very difficult to have a personal connection with a computer screen.

## Discussion

### Principal Findings

Our review revealed that the empowerment caregivers experienced from participating in a web-based psychoeducation program was built on knowledge sharing, individualized support from the program facilitator, and skill building to foster positive thoughts. This empowerment enables the active management of care activities. Our findings support previous studies that define empowerment for caregivers as a learning process that enables them to improve their coping capabilities by enhancing self-efficacy and self-determination, thereby creating more constructive relationships with the people surrounding them [35-37]. Self-efficacy is the belief that a person can complete tasks effectively when faced with stressors [38]. A positive outcome of self-efficacy is associated with cultivating positive thoughts and self-control [39]. According to the self-determination theory introduced by Ryan and Deci [40], people are motivated to learn to achieve their goals when they have a sense of self-control and self-efficacy and feel connected to other people. The carefully designed programs identified in our review reflect the development of these capabilities that empower caregivers in their caregiving role. Our finding on empowering learning is also in line with the study by Sakanashi and Fujita [36], in which empowering education programs for caregivers of people living with dementia included coping strategies, understanding the caregiver role, self-reflection, and quality information to enable the person to find autonomy and the capacity to take on the role.

We found that peer support through psychoeducation programs has a positive impact on caregivers’ experiences. Caring for people living with dementia is associated with social isolation because of demands from caregiving and dementia stigma [41]. Peer support provides caregivers with opportunities to communicate with others and share their experiences, which can potentially help them acquire new knowledge, build skills, develop resilience, and reduce caregiver burden [42-45]. The caregivers in this review valued peer support experiences, reflecting on the benefits they received. Research also shows that knowledge exchange through peer interactions can improve caregivers’ sense of self-efficacy [46] and reduce depressive symptoms [47]. In contrast, the absence of group learning and support may be associated with a low level of self-efficacy [48].

Our review revealed the caregivers’ preferences regarding the content of web-based psychoeducation programs. From our review, video components were preferred by caregivers as a means to facilitate a better understanding of the information presented. We found that caregivers were particularly touched by videos that portrayed real-life stories. The findings of our review also indicated that the relevance of information presented in pictures and text influenced caregivers’ experiences. This finding could be explained in the context of human cognitive function in processing information, in which visual stimuli, such as pictures, text, and videos, during focused attention are useful for learners to attain new knowledge [49,50]. However, the cognitive learning process is based on the condition that the information, or learning content, is relevant to learners [49]. A study that explored caregivers’ information needs and information-seeking behaviors indicated that the most frequently requested information was general information on dementia, care provision, self-care, and how to use available services [51]. A caregiver’s decision to access information depends on the quality and trustworthiness of the source [52]. Caregivers in this review valued learning content that facilitated reflection on their role and promoted self-care. In addition, our review found that caregivers’ learning needs were influenced by the stages of their dementia journey. Caregivers requested that information should be tailored to accommodate their differences, thereby enhancing their learning experience while avoiding mismatches between information and learning content. The information included in web-based programs should be tailored to the individual’s situation and address the individual’s needs, while simultaneously preventing information overload.

In this review, we identified that the technical design of a web-based psychoeducation program is another factor that influences caregivers’ learning experiences. The visual layout, structure, language used, functionality, and accessibility of the web-based program were important to the caregivers. Caregiver expectations in these aspects of program design within this review can be explained by how people sense and perceive the information displayed in a web-based program. The first step in human cognitive functioning for information processing occurs via the sensory system (ie, visual and audio), which filters out irrelevant information, notes the information that is of interest and relevance via short-term memory, and then lays down long-term memories [53]. According to Vu et al [50], website design needs to consider the user’s cognitive and physical capabilities. For example, older people will see contents on the screen more easily when the program design avoids the use of blue or green colors from the short-wavelength end of the visual spectrum and increases the resolution of screen contents [50]. The caregivers’ feedback on the web-based psychoeducation program design noted in our review reflects these recommendations.

Our review also identified various challenges for caregivers when using web-based psychoeducation programs and learning on the web. These challenges included, but were not limited to, caregiving demands, especially for those in the workforce, technical issues, and program design. In contrast to previous studies, our review did not identify caregivers’ concerns about the privacy and confidentiality of their information [54,55]. We found that although caregivers, especially those living in remote areas, perceived web-based psychoeducation programs as flexible, caregiving demands precluded many working caregivers from participating. Previous studies have found that web-based programs can support working caregivers to achieve a balance between work and caregiver demands, supporting them through web-based peer interactions that save both time and money [55,56], but this does require an individual’s resolution. According to West and Hogan [57], regular support group attendance was associated with members’ perception of support from the group, subjective well-being, compromises they made, and care responsibilities. Moreover, according to our review, using a web-based program depends on an individual’s perception of how useful it is to address their needs. Research has identified that working caregivers report lower carer confidence compared with nonworking caregivers, indicating the need for additional support to build their skills and confidence [58]. However, the educational support programs reviewed here do not necessarily reflect this. A flaw noted in this review was that most programs were not available after the completion of the study, despite participants wanting to revisit some of the information. A previous study suggested that program usefulness depended on whether the function and cost met individual needs [59]. These factors potentially influence caregivers’ feelings about web-based programs’ usefulness in the long term.

It is important to consider group dynamics if peer interactions are included in a program. Previous studies have focused more on the positive aspects of support groups, with negative experiences rarely discussed. A forum was convened in 1 study to ascertain barriers to successful web-based group meetings and made recommendations, for example, that groups be arranged according to the similarity of caregivers’ experience, have clear meeting agendas, and consider participants’ diversity [60]. Other studies showed that the positive impact of support groups depended on peer interactions and how well groups were organized [57,61]. The caregivers in our review expressed concerns about poor peer interaction, lack of discussion topics, and lack of equal opportunities to contribute during group meetings. This highlights the importance of a trained facilitator leading a caregiver support group.

As identified in multiple studies [55,60], technical difficulties accessing a program, such as a poor internet connection, challenge the use of web-based programs, as does an individual’s confidence and computer skills [62,63]. In our review, most participants felt positive about web-based psychoeducation programs; but to meet a broader audience, programs must consider the caregiver population that may not be technically savvy.

### Recommendations

High-quality and carefully designed web-based psychoeducation programs offer positive experiences to informal caregivers of people living with dementia. To meet broader caregiver education and support needs, program designers should consider the following recommendations (Multimedia Appendix 7). First, the learning content and information provided must be tailored to caregivers’ learning needs. This can be achieved by encouraging caregivers to self-diagnose their learning needs and select relevant sections. Second, web-based psychoeducation programs should include components to facilitate social connectedness among caregivers so that they can share their experiences and help each other. Third, having program facilitators who are trained health or social care professionals is imperative for engaging caregivers in the program and providing individualized support. Fourth, programs should integrate multimodality teaching materials, such as text, videos, discussion boards, and supporting group meetings, to attract learners at the cognitive information processing level. Fifth, asynchronized web-based learning and teaching are recommended to accommodate a broader audience, especially working caregivers. Sixth, the program content should be developed based on an education needs analysis of caregivers. Program providers should conduct ongoing evaluations of the quality and relevance of the information presented to ensure caregivers’ confidence in the program, thereby enhancing its utilization. Seventh, initial training and ongoing technical support for caregivers are required when implementing web-based psychoeducation programs. A program should be accompanied by hard copy instructions to support caregivers when technical issues arise. Finally, most psychoeducation research has focused on program effectiveness. Future research should also focus on informal caregivers’ experiences of using web-based psychoeducation programs to increase utilization.

### Limitations

The main strength of this review is the rigorous adherence to the JBI systematic review and meta-aggregation protocol to minimize bias during the process. However, this review has a few limitations. First, only 9 articles were included; this is an indicator that research evidence from qualitative studies is limited. Second, this review was based on database searches in 3 languages: English, Chinese, and Arabic. Therefore, a bias exists in the selection of studies. Despite the primary effort to review studies in Chinese and Arabic, the lack of diverse evidence from different contexts in non-English studies is apparent. The caregivers’ experiences identified in this review may not be representative of a wider culturally and linguistically diverse population. Transferability to similar contexts in qualitative research needs to be confirmed by the reader.

### Conclusions

This is the first comprehensive systematic review to synthesize qualitative studies on dementia caregivers’ experiences in web-based psychoeducation programs in a global context. The findings contribute to new knowledge about caregivers’ learning experiences, including interactions with peers, learning content, program technical design, and challenges encountered in web-based programs. The synthesized findings confirmed that multiple factors affected informal caregivers’ experiences. The enabling factors most often mentioned included the programs’ quality and relevancy, support received, relevance to individual caregivers’ needs, flexibility in delivery, and ability to connect to other caregivers and program facilitators without time and space restrictions. The impeding factors included caregiving demands, poor program performance (eg, internet connection), and the inability to meet individual caregiver’s needs (eg, their caring situation) or preferences (eg, for a paper-based program).

## Appendix

Multimedia Appendix 1 Keywords.

Multimedia Appendix 2 Search strategies.

Multimedia Appendix 3 Findings and illustrations.

Multimedia Appendix 4 PRISMA (Preferred Reporting Items for Systematic Reviews and Meta-Analyses) 2020 item checklist.

Multimedia Appendix 5 Table results of metasynthesis.

Multimedia Appendix 6 The ConQual summary of findings.

Multimedia Appendix 7 The meta-aggregation flowchart.

## Abbreviations

JBI: Joanna Briggs Institute
PRISMA: Preferred Reporting Items for Systematic Reviews and Meta-Analyses
