# Supplementary material for: The Experiences of Informal Caregivers of People With Dementia in Web-Based Psychoeducation Programs: Systematic Review and Metasynthesis
Source: JMIR Aging. 2023 May 29;6:e47152. doi: 10.2196/47152 (PMC10262022; doi:10.2196/47152)
Supplement: Multimedia Appendix 1 [file aging_v6i1e47152_app1.docx]

**Appendix 1 Keywords**

| Population | Interest | Context | Research type |
| --- | --- | --- | --- |
| Informal caregivers of people living with dementia | Online dementia carer education  Experience | Homecare settings | Qualitative and Mixed Methods |
| Carer OR Caregiver OR carer taker OR family OR friend OR spouse OR adult children or acquaintance OR neighbour or neighbour OR (home nursing  people with dementia) OR Alzheimer’s disease OR Mild Cognitive Impairment | Internet OR online or e-health OR telecomputing OR tech or health OR computers OR software OR electronic OR digital  Intervention OR program OR course OR psychoeducation OR training OR support OR cognitive therapy OR psychological OR platform OR e-learning or telemedicine OR telehealth OR application OR interface OR APP  Views, experiences, opinions, attitudes, perceptions, beliefs, feelings, knowledge, OR  understanding |  |  |
