# Supplementary material for: The Experiences of Informal Caregivers of People With Dementia in Web-Based Psychoeducation Programs: Systematic Review and Metasynthesis
Source: JMIR Aging. 2023 May 29;6:e47152. doi: 10.2196/47152 (PMC10262022; doi:10.2196/47152)
Supplement: Multimedia Appendix 2 [file aging_v6i1e47152_app2.docx]

**Appendix 2 Search strategies**

Medical Literature Analysis and Retrieval System Online (MEDLINE)

| 1 | carers. mp. or exp Caregivers/ | | 49222 | 7.7.2021 |
| --- | --- | --- | --- | --- |
| 2 | (Carer* or caregiver* or carer taker*or caretaker* or spouse or adult children or acquaintance* or neighbor* or neighbour* or home nursing).mp. [mp=title, abstract, original title, name of substance word, subject heading word, floating sub-heading word, keyword heading word, organism supplementary concept word, protocol supplementary concept word, rare disease supplementary concept word, unique identifier, synonyms] | 242676 | |  |
| 3 | (dementia*or Alzheimer* or "Mild Cognitive Impairment").ti,ab. | 18482 | |  |
| 4 | exp Dementia/ | 177047 | |  |
| 5 | exp Dementia/ or exp Alzheimer Disease/ | 177047 | |  |
| 6 | (Internet or online or e-health or e-learning or telecomputing or tech or health or computers or software or electronic or digital).ti,ab. | 2673306 | |  |
| 7 | Computer-Assisted Instruction/ | 12140 | |  |
| 8 | (Intervention* or program* or course* or psychoeducation* or training* or support* or platform* or e-learning or telemedicine or telehealth or application* or interface).ti,ab. | 5418314 | |  |
| 9 | (View* or experience* or opinion*, attitude* or perception* or belief* or feeling* or knowledge or understanding*).mp. [mp=title, abstract, original title, name of substance word, subject heading word, floating sub-heading word, keyword heading word, organism supplementary concept word, protocol supplementary concept word, rare disease supplementary concept word, unique identifier, synonyms] | 3470785 | |  |
| 10 | 1 or 2 | 242676 | |  |
| 11 | 3 or 4 or 5 | 186208 | |  |
| 12 | 6 or 7 | 2679543 | |  |
| 13 | 8 and 9 and 10 and 11 and 12 | 1207 | |  |
| 14 | limit 13 to the English language | 1142 | |  |

Cumulative Index to Nursing and Allied Health Literature (CINAHL)

| 1 | TX Carer* or caregiver* or "carer taker*” or caretaker* or spouse or "adult children" or acquaintance* or neighbor* or neighbour* or "home nursing" | | 140,276 | 7.7.2021 |
| --- | --- | --- | --- | --- |
| 2 | TX (MH "Mild Cognitive Impairment") OR (MH "Alzheimer's Disease") OR "dementia*or Alzheimer* or "Mild Cognitive Impairment"" | 34,046 | |  |
| 3 | TX "Internet or online or e-health or e-learning or telecomputing or tech or health or computers or software or electronic or digital" OR (MH "Learning Health System") OR (MH "World Wide Web") OR (MH "Online Education") OR (MH "World Wide Web Applications") | 82,975 | |  |
| 4 | (MH "Online Education") OR "Intervention* or program* or course* or psychoeducation* or training* or support* or platform* or e-learning or telemedicine or telehealth or application* or interface" OR (MH "Education, Non-Traditional") OR (MH "Programmed Instruction") OR (MH "Adult Education") | 13,009 | |  |
| 5 | S1 AND S2 AND S3 AND S4 | 1 | |  |

Web of Science

| 1 | Carer* or caregiver* or "carer taker*”or caretaker* or spouse or "adult children" or acquaintance* or neighbor* or neighbour* or "home nursing" (All Fields) | | 564,621 | 7.7.2021 |
| --- | --- | --- | --- | --- |
| 2 | ALL=(dementia*or Alzheimer* or "Mild Cognitive Impairment" ) | 34,042 | |  |
| 3 | ALL=(Internet or online or e-health or e-learning or telecomputing or tech or health or computers or software or electronic or digital) | 16,544,485 | |  |
| 4 | ALL=(Intervention* or program* or course* or psychoeducation* or training* or support* or platform* or e-learning or telemedicine or telehealth or application* or interface ) | 13,009 | |  |
| 5 | ALL=(View* or experience* or opinion*, attitude* or perception* or belief* or feeling* or knowledge or understanding*) | 6,923,690 | |  |
| 6 | (((((ALL=(View* or experience* or opinion*, attitude* or perception* or belief* or feeling* or knowledge or understanding*)) AND #5) AND #4) AND #3) AND #2) AND #1 | 314 | |  |

Scopus

| 1 | TITLE-ABS-KEY ( carer* OR caregiver* OR "carer taker*" OR caretaker* OR spouse OR "adult children*" OR acquaintance* OR neighbor* OR neighbour* OR "home nursing" ) | | 755,407 | 7.7.2021 |
| --- | --- | --- | --- | --- |
| 2 | TITLE-ABS-KEY ( dementia* OR alzheimer* OR "mild cognitive impairment" ) | 365,308 | |  |
| 3 | TITLE-ABS-KEY ( internet OR online OR e-health OR e-learning OR telecomputing OR tech OR health OR computers OR software OR electronic OR digital ) | 13,187,156 | |  |
| 4 | TITLE-ABS-KEY ( intervention* OR program* OR course* OR psychoeducation* OR training* OR support* OR platform* OR e-learning OR telemedicine OR telehealth OR application* OR interface ) | 18,855,762 | |  |
| 5 | TITLE-ABS-KEY ( view* OR experience* OR opinion* OR attitude* OR perception* OR belief* OR feeling* OR knowledge OR understanding* ) | 9,529,135 | |  |
| 6 | ( TITLE-ABS-KEY ( internet OR online OR e-health OR e-learning OR telecomputing OR tech OR health OR computers OR software OR electronic OR digital ) ) AND ( TITLE-ABS-KEY ( intervention* OR program* OR course* OR psychoeducation* OR training* OR support* OR platform* OR e-learning OR telemedicine OR telehealth OR application* OR interface ) ) AND ( TITLE-ABS-KEY ( view* OR experience* OR opinion* OR attitude* OR perception* OR belief* OR feeling* OR knowledge OR understanding* ) ) AND ( TITLE-ABS-KEY ( dementia* OR alzheimer* OR "Mild Cognitive Impairment" ) ) AND ( TITLE-ABS-KEY ( carer* OR caregiver* OR "carer taker*" OR caretaker* OR spouse OR "adult children*" OR acquaintance* OR neighbor* OR neighbour* OR "home nursing" ) ) | 3923 | |  |
| 7 | LIMITED 6 to English | 3706 | |  |

**Chinese data-base search strategies**

**Key words**

| 研究对象 | 照顾者or非正式照顾者or照护者or家属or家人or配偶or妻子or朋友or儿女or儿子or女儿or子女or亲属or亲戚or邻居or照料者  痴呆or老年痴呆or血管性痴呆or额颞叶性痴呆or帕金森痴呆or失智症or认知症or认知障碍or阿尔茨海默or阿尔兹海默or Alzheimer's or AD or轻度认知障碍or MCI |
| --- | --- |
| 干预 | 互联网or网络or网站or在线or计算机or远程or手机or电脑or笔记本or电子or软件or APP or微信or QQ or移动设备or视频or公众号or媒体or小程序  干预or治疗or训练or疗法or项目or课程or教育or心理教育or心理or培训or支持or信息平台or医疗平台or平台or移动医疗or远程医疗or应用or界面or设备 |

**万方(Wanfang)**

| **检索式** | | **检索量** | **检索时间** |
| --- | --- | --- | --- |
| **1** | 主题:("痴呆"or"老年痴呆"or"血管性痴呆"or"额颞叶性痴呆"or"帕金森痴呆"or"失智症"or"认知症"or"认知障碍"or"阿尔茨海默"or"阿尔兹海默"or"Alzheimer's"or"AD"or"轻度认知障碍"or"MCI") | 655979 | 2021.07.12 |
| **2** | 主题:("照顾者"or"非正式照顾者"or"照护者"or"家属"or"家人"or"配偶"or"妻子"or"朋友"or"儿女"or"儿子"or"女儿"or"子女"or"亲属"or"亲戚"or"邻居"or"照料者") | 857757 |  |
| **3** | 主题:("互联网"or"网络"or"网站"or"在线"or"计算机"or"远程"or"手机"or"电脑"or"笔记本"or"电子"or"软件"or"APP"or"微信"or"QQ"or"移动设备"or"视频"or"公众号"or"媒体"or"小程序") | 10709683 |  |
| **4** | 主题:("干预"or"治疗"or"训练"or"疗法"or"项目"or"课程"or"教育"or"心理教育"or"心理"or"培训"or"支持"or"信息化平台"or"医疗平台"or"移动医疗"or"平台"or"远程医疗"or"应用"or"界面"or"设备") | 31649708 |  |
| **5** | 1 and 2 and 3 and 4 | 205 |  |

**中国知网(CNKI)**

| **检索式** | | **检索量** | **检索时间** |
| --- | --- | --- | --- |
| **1** | SU %=‘痴呆’+‘老年痴呆’+‘血管性痴呆’+‘额颞性痴呆’+‘帕金森痴呆’+‘失智症’+‘认知症’+‘认知障碍’+‘阿尔茨海默’+‘阿尔兹海默’+‘AD’+‘轻度认知障碍’+‘MCI’+‘Alzheimer’s’ | 106,316 | 2021.07.12 |
| **2** | KY=‘痴呆’+‘老年痴呆’+‘血管性痴呆’+‘额颞性痴呆’+‘帕金森痴呆’+‘失智症’+‘认知症’+‘认知障碍’+‘阿尔茨海默’+‘阿尔兹海默’+‘AD’+‘轻度认知障碍’+‘MCI’+‘Alzheimer’s’ | 22,901 |  |
| **3** | AB=‘痴呆’+‘老年痴呆’+‘血管性痴呆’+‘额颞性痴呆’+‘帕金森痴呆’+‘失智症’+‘认知症’+‘认知障碍’+‘阿尔茨海默’+‘阿尔兹海默’+‘AD’+‘轻度认知障碍’+‘MCI’+‘Alzheimer’s’ | 119,418 |  |
| **4** | SU %='照顾者'+'非正式照顾者'+'照护者'+'家属'+'家人'+'配偶'+'妻子'+'朋友'+'儿女'+'儿子'+'女儿'+'子女'+'亲属'+'亲戚'+'邻居'+'照料者' | 199,434 |  |
| **5** | KY='照顾者'+'非正式照顾者'+'照护者'+'家属'+'家人'+'配偶'+'妻子'+'朋友'+'儿女'+'儿子'+'女儿'+'子女'+'亲属'+'亲戚'+'邻居'+'照料者' | 6,653 |  |
| **6** | AB='照顾者'+'非正式照顾者'+'照护者'+'家属'+'家人'+'配偶'+'妻子'+'朋友'+'儿女'+'儿子'+'女儿'+'子女'+'亲属'+'亲戚'+'邻居'+'照料者' | 436,295 |  |
| **7** | SU %=‘互联网’+’网络’+’网站’+’在线’+’计算机’+’远程’+’手机’+’电脑’+’笔记本’+’电子’+’软件’+’APP’+’微信’+’QQ’+’移动设备’+’视频’+’公众号’+’媒体’+’小程序’ | 3,891,934 |  |
| **8** | KY=‘互联网’+’网络’+’网站’+’在线’+’计算机’+’远程’+’手机’+’电脑’+’笔记本’+’电子’+’软件’+’APP’+’微信’+’QQ’+’移动设备’+’视频’+’公众号’+’媒体’+’小程序’ | 5,237 |  |
| **9** | AB=‘互联网’+’网络’+’网站’+’在线’+’计算机’+’远程’+’手机’+’电脑’+’笔记本’+’电子’+’软件’+’APP’+’微信’+’QQ’+’移动设备’+’视频’+’公众号’+’媒体’+’小程序’ | 4,909,096 |  |
| **10** | SU %=‘干预’+’治疗’+’训练’+’疗法’+’项目’+’课程’+’教育’+’心理教育’+’心理’+’培训’+’支持’+’信息化平台’+’医疗平台’+’移动医疗’+’平台’+’远程医疗’+’应用’+’界面’+’设备’ | 12,631,938 |  |
| **11** | KY=‘干预’+’治疗’+’训练’+’疗法’+’项目’+’课程’+’教育’+’心理教育’+’心理’+’培训’+’支持’+’信息化平台’+’医疗平台’+’移动医疗’+’平台’+’远程医疗’+’应用’+’界面’+’设备’ | 48 |  |
| **12** | AB=‘干预’+’治疗’+’训练’+’疗法’+’项目’+’课程’+’教育’+’心理教育’+’心理’+’培训’+’支持’+’信息化平台’+’医疗平台’+’移动医疗’+’平台’+’远程医疗’+’应用’+’界面’+’设备’ | 16,708,134 |  |
| **13** | 1 OR 2 OR 3 | 168,943 |  |
| **14** | 4 OR 5 OR 6 | 522,700 |  |
| **15** | 7 OR 8 OR 9 | 5,983,473 |  |
| **16** | 10 OR 11 OR 12 | 19,939,887 |  |
| **17** | 1 AND 2 AND 3 AND 4 | 344 |  |

**超星期刊(Chao xing)**

| **检索式** | | **检索量** | **检索时间** |
| --- | --- | --- | --- |
| **1** | Su=痴呆\|失智\|认知症\|认知障碍\|阿尔茨海默\|阿尔兹海默\|Alzheimer’s\|AD\|轻度认知障碍\|MCI | 218,390 | 2021.07.12 |
| **2** | Su=照顾者\|照护者\|家属\|家人\|配偶\|朋友\|儿女\|儿子\|女儿\|子女\|亲属\|亲戚\|邻居\|照料者 | 1,899,488 |  |
| **3** | Su=互联网\|网络\|网站\|在线\|计算机\|远程\|手机\|电脑\|笔记本\|电子\|软件\|APP\|微信\|QQ\|移动设备\|视频\|公众号\|媒体\|小程序 | 9,509,204 |  |
| **4** | Su=干预\|治疗\|训练\|疗法\|项目\|课程\|教育\|心理教育\|心理\|培训\|支持\|信息平台\|医疗平台\|平台\|移动医疗\|远程医疗\|应用\|界面\|设备 | 34,905,180 |  |
| **5** | 1 AND 2 AND 3 AND 4 | 447 |  |

**维普（VIP）**

| **检索式** | | **检索量** | **检索时间** |
| --- | --- | --- | --- |
| **1** | M=痴呆 or 老年痴呆 or 血管性痴呆 or 额颞叶痴呆 or 帕金森痴呆 or 失智症 or 认知症 or 认知障碍 or 阿尔茨海默 or 阿尔兹海默  or AD or 轻度认知障碍 or MCI or Alzheimer | 82,898 | 2021.07.12 |
| **2** | M=照顾者 or 非正式照顾者 or 照护者 or 家属 or 家人 o r配偶 or 妻子 or 朋友 or 儿女 or 儿子 or 女儿 or 子女 or 亲属 or 亲戚 or 邻居 or 照料者 | 265,559 |  |
| **3** | M=互联网 or 网络 or 网站 or 在线 or 计算机 or 远程 or 手机 or 电脑 or 笔记本 or 电子 or 软件 or APP or 微信 or QQ or 移动设备 or 视频 or 公众号 or 媒体 or 小程序 | 4,112,128 |  |
| **4** | M=干预 or 治疗 or 训练 or 疗法 or 项目 or 课程 or 教育 or 心理教育 or 心理 or 培训 or 支持 or 信息平台 or 医疗平台 or 平台 or 移动医疗 or 远程医疗 or 应用 or 界面 or 设备 | 14,057,408 |  |
| **5** | 1 AND 2 AND 3 AND 4 | 9 |  |
