# Supplementary material for: The Experiences of Informal Caregivers of People With Dementia in Web-Based Psychoeducation Programs: Systematic Review and Metasynthesis
Source: JMIR Aging. 2023 May 29;6:e47152. doi: 10.2196/47152 (PMC10262022; doi:10.2196/47152)
Supplement: Multimedia Appendix 3 [file aging_v6i1e47152_app3.docx]

**Appendix 3 Findings and illustrations**

Brennan, P. F., Moore, S. M., & Smyth, K. A. (1991). ComputerLink: electronic support for the home caregiver. ANS Adv Nurs Sci, 13(4), 14-27. <https://doi.org/10.1097/00012272-199106000-00004>

| Findings | Illustration from the study | Evidence |
| --- | --- | --- |
| Peer support: opportunity for caregivers to interact with each other | There are frequent statements of encouragement and support among caregivers for example: ‘my husband is in the middle stages of the disease and I would like some suggestions on how to occupy his time…’ ‘Dorothy I also have a problem with my wife who likes to walk and gets bored…’ ‘ Hi this is Sue. I noticed a reply to idle Time,…’ (p.21) | Unequivocal |
| Feeling of being supported by program providers | ‘I am a new member who wonders how many of you feel abandoned by your friends…’ ‘Mrs C, you have brought up a question that many people have expressed to me.’(p.21) | Unequivocal |
| Program designs: practical and relevant | Most used content in the program:  research on new medications; obtaining an identification bracelet; choosing a day-care centre; choosing a nursing home; local education programs; Topics relate to behaviour management; Topics on caregiver coping skills include how to manage anger, lack of patience, feeling of rejection by family and friends, loneliness, fatigue, relationship problems with care recipient, change, frustration, working outside the home while caregiving; Q&A section for various concerns caregivers had. (p.24-25) | Credible |

Duggleby, W., Ploeg, J., McAiney, C., Fisher, K., Jovel Ruiz, K., Ghosh, S., Peacock, S., Markle-Reid, M., Williams, A., Triscott, J., & Swindle, J. (2019). A comparison of users and nonusers of a web-based intervention for carers of older persons with Alzheimer disease and related dementias: Mixed methods secondary analysis. J Med Internet Res, 21(10), e14254-e14254. <https://doi.org/10.2196/14254>

| Findings | Illustration from the study | Evidence |
| --- | --- | --- |
| Difficulties with site access and navigation | “...my internet connection at home is poor—I live in a rural area.” (p.9) | Unequivocal |
| Low level of computer literacy contributed to access difficulties | “No, it—it’s, uh, as far as the computer is concerned, it’s the—the operator of it that’s at fault.”; another said: “Um, well, I get frustrated at myself when, you know, I’m working on the website…” (p.9) | Unequivocal |
| Too stressed to take part | “I’m extremely stressed with taking care of my wife, and so I lost the email with login instructions.”  “he kept interrupting me. Then, I couldn’t find where I left off to continue...” (p.9) | Unequivocal |
| Insufficient time for program participation | “...and I got to admit that it was, uh, something that, uh, I didn’t go onto too much, just strictly because of all the other things that were—were going on this past month.” (p. 9)  “Well, um, I just finished reading it, and—and—and then, I had to go off because I had to go help my husband.” (p.9)  “...[I] work full time early morning to late evening...and at the end of the day, I don’t have the energy or time to go on the computer.” (p.9) | Unequivocal |
| Personal preference for hard copy | “...Sometimes, you actually have to have something printed in front of you, uh, and I’m better off—I’m better with paper. In some instances, to sit and reflect, I’m not really good at what—I’m not really one of those people who can do it all online.” (p.10). | Unequivocal |
| Personal preference of actual social contact with others | “I think—I think I know—and this is [chuckles]—this isn’t specific to this Toolkit, but it sort of relates to it: um, I think I’m the kind of person who gets a lot more out of, you know, actual social interaction around something (p.10). | Unequivocal |

Fowler, C. N., Haney, T., & Lemaster, M. (2016). Helping Dementia Caregivers Through Technology. Home Healthc Now, 34(4), 203-209. <https://doi.org/10.1097/NHH.0000000000000372>

| Findings | Illustration from the study | Evidence |
| --- | --- | --- |
| Peer support: opportunity for caregivers to interact with each other | “I think it was beneficial to me to read other people’s stories and interact with them online. (p.208 ).  It wasn’t so much from a perspective of, ‘Oh, I’m not out here alone,’ kind of thing but just to be able see what other people’s stories were like, how others were handling things and seeing how people interacted with each other. That medium was really valuable.” (p.208). | Unequivocal |
| Feeling of being supported by program providers | ‘The site really helped me through tough times. Having two small children at home and my mom with advanced stage of Alzheimer’s disease was very difficult. I appreciate everything the team did to help me through that…Being a part of the study at that time in my life really helped me cope with difficult family issues and decisions.” (p.208). | Unequivocal |
| Streamlined program provided positive learning experience | “I was a little intimidated with it at first but then I got on and it worked very smoothly, you know, the way it was supposed to and it made the experience kind of fun.” (p.207). | Unequivocal |
| Accessibility: enhanced outreach of program | The idea was exceptional. I wish there were more people who participated in the study. It has great potential for community support (p.207).  “Reach a broader audience. I’m not a tech person and I’m not sure what I could contribute myself, but I felt good about the project. Maybe advertise on community networks/apps such as ‘Next Door.’ There are so many people that stay home with children or elderly folks – those are the people that would benefit from it. I was pleased with the study and was honoured to participate.” (p.208). | Unequivocal |

Gaugler, J. E., Hobday, J. V., Robbins, J. C., & Barclay, M. P. (2015). CARES((R)) Dementia Care for Families: Effects of Online, Psychoeducational Training on Knowledge of Person-Centered Care and Satisfaction. J Gerontol Nurs, 41(10), 18-24. <https://doi.org/10.3928/00989134-20150804-61>

| Findings | Illustration from the study | Evidence |
| --- | --- | --- |
| Hearing all perspectives empowers caregivers | …the examples and the stories of families who live with Alzheimers were very informative and gave me comfort that I, too, can do this (p.22).  I would tell them (and already have told several people from my Alzheimer’s [sic] support group) that this program gives real-life instances of issues that arise and suggestions for working through them. The program also gives the perspective of those living with the disease—something I am clueless about. I feel empowered because of the information I’ve been given. (p.22). | Unequivocal |
| Videos: related to disease progression | I really liked the videos that showed the progression of the disease in the early, middle, and late stages of the disease. For example: the making coffee and taking a bath example. I also liked the driving example, too, in relation to the different parts of the brain and how they are affected. As a 22 caregiver, it really helps me get an idea of how my family member is going to progress (p.21). | Unequivocal |
| Videos: poor visual display quality | A few technical and display issues. Video segments were displayed smaller than text window…had to go into settings to increase video screen size. Audio was cut short on several of the slides (p.22). | Unequivocal |
| Relevant content: Information for the start of the caregiving journey | ’I found all of this training very helpful and well thought out…I have been  caring for my grandmother full-time in my home for the past 9 months…this training would have been very helpful if it were available to us at the start of our journey. I did hours of Internet [sic] research to gather the many tips and strategies presented in the training, and they work (p.22). | Unequivocal |
| Program designs: practical and relevant | I consider myself an even keeled, compassionate person. Yes, sometimes I get frustrated and have to leave the room to keep from saying something I might later regret. This training program showed me ANOTHER WAY, and I didn’t even know there was one (p.22). | Unequivocal |
| Program design: negative case scenario | I didn’t like the ending. I found it very sad to be left with the vision of the dear man peeling bananas. You could have chosen something a bit more uplifting…I felt that they had all come to terms and made the best of it—and so can we!!! (p.22) | Unequivocal |
| Accessibility: enhanced reach of program | I would say that this is a great program for the journey involved in an Alzheimer’s diagnosis, and that this is useful throughout the progression for all stages and for different roles involved in the diagnosis. If this program were available for purchase on DVD, I would consider buying and sharing with family members to educate them and help them to help me care for my mother | Unequivocal |

Halbach, T., Solheim, I., Ytrehus, S., & Schulz, T. (2018). A mobile application for supporting dementia relatives: A case study (Vol. 256, pp. 839-846).

| Findings | Illustration from the study | Evidence |
| --- | --- | --- |
| Inspiring texts | but also the self-reflection prose related to these because the text was inspiring and resulted in a more efficient learning experience. (p.844) | Unequivocal |
| Video: relevant structure and content | All lectures were rated as being clear and comprehensive… The lectures were judged as being “quite relevant” and “very relevant” (Halbach et al., 2018, p.843)  The informants found the structuring of lectures, including basic and extended versions, takeaways, and local information (quite or very) useful and gave the same judgement for the “Getting started” lecture, and the font size. (p.843) | Unequivocal |
| Videos: poor audio quality | An unfamiliar machine voice and a “sharp voice” in videos, which is most likely due to a quite high compression rate for audio in videos. (p.843)  A poor pronunciation of medical terms with artificial voices, which is a real problem in many low-level text-to-speech solutions. (p.843)  The reading aloud option was generally viewed as less successful. Some said they did not need this option, others found the (computer) voice too monotonous and hard to follow. (p.843) | Credible |
| Videos: promotes understanding | When it comes to content, the participants (including health workers) liked videos in particular, (p.844).  The informants found the videos to be (quite and very) useful and commented on “too few” in their comments. (p.843) | Credible |
| Relevant content: accommodating caregivers learning needs | It is useful to distinguish between basic and in-depth information. It was convenient for the participants to read the short basic information first and, if needed, to look more closely at in-depth material later on when there was more time. (p.844) | Credible |
| Relevant content: applicable for other family members | Several persons also commented positively on the information designed for and aimed at children, but also advised that children should have easy access to all other types of information in the app, too. (p.844) | Credible |
| Program designs: practical and relevant | A more hands-on character of some lectures in form of examples and practical advice was requested. However, also realism was asked for, as one of the patients in the video was “too easy to distract”. (p.843) | Credible |
| Program designs: need for locally relevant information | The informants recommended more local information, but at the same time they desired this to be easily available in the app rather than to be redirected to the municipality’s web page. (p.844) | Credible |
| Program design: lack of comprehensiveness | It was a known issue that the 23 lectures were not covering the entire area, and this was also remarked on with several participants mentioning missing topics and in-depth information. (p.843) | Credible |
| Program designs: user friendly text | The limited amount of text per page and short paragraphs worked very well for these participants. (p.844) (Halbach et al., 2018)  We had set the font size to values larger than what is usual in apps of this kind, and the participants liked this because it made, as they said, the app more accessible. (p.844) | Credible |
| Program design: Learning unit structure | In general, the informants were satisfied with the app and found it quite useful. The structure of learning units / lectures worked very well (p.844). | Credible |
| Program designs: Quality of grammar | Quizzes and “text to speech” got a neutral rating, and one particularly critical person pointed to what she viewed as bad grammar and poor language. (p.843) | Credible |
| Program design Quizzes not suitable | Most of the participants found the quiz option less attractive. The option might be useful for children, they commented, but advised to remove it from the app. (p.844) | Credible |
| Content design: relevant and targeted information | For a few participants the content was known from before, which resulted in a lower rating usefulness rating than what was the case in reality. (p.843) | Credible |

Hattink, B., Droes, R.-M., Sikkes, S., Oostra, E., & Lemstra, A. W. (2016). Evaluation of the Digital Alzheimer Center: Testing Usability and Usefulness of an Online Portal for Patients with Dementia and Their Carers. *JMIR Res Protoc*, *5*(3), e144-e144. https://doi.org/10.2196/resprot.5040

| Findings | Illustration from the study | Evidence |
| --- | --- | --- |
| Feeling of being supported by program providers | Interviewed participants specified that it was “very helpful—it really helps me in staying at home by myself” and that it “should certainly be continued in the future.” | Unequivocal |
| Technical issues precluded engagement | The main reasons they indicated for not using the DAC are: Technical or computer issues , Miscellaneous (eg, “I don’t like the Internet”) | Credible |
| Insufficient time for program participation | The main reasons they indicated for not using the DAC are: No time | Credible |
| Program designs: tailored to meet individual needs | “not yet useful enough,” although they later indicated that they expected this would change by “adding more personalization [options].”(p.8) | Unequivocal |
| Program design: easy to navigate | you can find all the information you might need” and “you can easily show this information to others.”’(p.9) | Unequivocal |
| Program design: flexibility of learning | “you can check this information anytime, even in the middle of the night.” (p.9) | Unequivocal |

Kovaleva, M., Blevins, L., Griffiths, P. C., & Hepburn, K. (2019). An Online Program for Caregivers of Persons Living With Dementia: Lessons Learned. J Appl Gerontol, 38(2), 159-182. <https://doi.org/10.1177/0733464817705958>

| Findings | Illustration from the study | Evidence |
| --- | --- | --- |
| Peer support: opportunity for caregivers to interact with each other | I felt a connection with every person and believe that I learned something from each participant” (Caucasian daughter, age 66). (p.168).  All caregivers stated that they felt listened to and could get answers for their questions: One of the very helpful parts of the chats was to have positive feedback from the teachers. I don’t think caregivers get very many “good job on that” . . . comments. It is easy to know when we mess up . . . hard to know that we did it well. (Caucasian wife, age 57) (p.168).  “Wonderful class, we need more . . . You have this group and they bond over 6-7 weeks, there is so much more to learn out there” (Caucasian wife, age 60). (p.170)  “[The] group got closer towards the end; people shared some powerful things; we did not have the time to process those powerful things” (Caucasian daughter, age 61). (p.171 ) | Unequivocal |
| Peer support: poor group interactions | An African American niece (age 47) commented: “It is a lot harder to connect with people, a lot easier not to stay focused on the topic, you can get distracted, play games on your phone.” This caregiver was in a group with others who were relatively younger (ages 53, 55, 61).(p.168)  A Caucasian daughter (age 49) noted, “People that did not appear to be paying attention and you could see them doing other things. That was a little distracting for me.” (p.168) | Unequivocal |
| Peer support: Lack of equal opportunity to contribute | “I don’t think I ever was clear on what the purpose of the weekly calls was because I never really walked away from the calls with much practical advice on what to do next.” She did not find videoconferences engaging or facilitating for each caregiver to have equal amounts of time to speak. (p.172) | Unequivocal |
| Peer support: More interaction and discussions required | “More interaction and discussion among the participants to balance out the information that is clinically-based, integrate both perspectives to just get different views on how other people cope.” (p.171)  “A one-on one session should be offered’. (p.171) | Unequivocal |
| Peer support: Reduced feelings of isolation | For me it was a lifesaver . . . seeing all those people from all around the country . . . they are not really handling it any better than I . . . I don’t feel so alone in spirit. (Caucasian wife, age 69) (p.167) | Unequivocal |
| Peer support: remaining in contact post-program | Several caregivers expressed willingness to remain in contact with others after Tele-Savvy conclusion. Facilitators asked for participants’ permission to have their contact information shared and, when such permission was granted, provided it to other caregivers. (p.168) | Unequivocal |
| Peer support: Preference for longer duration | “Make it longer, make it longer, make it longer. I cannot say it quite enough . . . Just a few things these professors [said], how they would listen, it was just a gift” (Caucasian daughter-in-law, age 49). Despite apparent novelty of videoconferences (no one indicated their experience with videoconferencing), they were valued: “I just feel like [we had an] amazing time—we could have gone for 3 hours” (Caucasian daughter-in-law, age 49). (p.171) | Unequivocal |
| Online class: like a real classroom | “At first I was . . . this is not gonna work; I’m 60 years old. It really worked, I loved going to school online, I thought I was in a real class—I’m talking a real classroom” (Caucasian wife, age 60). (p.168) | Unequivocal |
| Difficulties with site access and navigation | Problems during videoconferences (e.g., poor Internet connection, slow sound and video transmission, and insufficient instructions on joining videoconferences) affected connectedness: “When things went well [with technology], I definitely felt connected” (Caucasian wife, age 57). | Unequivocal |
| Low level of computer literacy contributed to access difficulties | Some caregivers noted that others struggled to follow some directions (e.g., not muting their microphone when others were speaking to prevent interference with background noise) and needed to be better aligned relative to their webcam and sit in a position with good lighting. (p.169) | Unequivocal |
| Personal preference of actual social contact with others | “It would have been better to absorb the content in a group setting, person to person . . . very difficult to have a personal connection with a computer screen.” (p.168)  a few younger caregivers and those who were employed outside of home (Caucasian son, age 53; African American niece, age 47) indicated a preference for a classroom experience. (p.168)  She commented that similar online programs may not fit older caregivers: I would recommend it to people who are tech savvy, but I wouldn’t recommend it to someone like my mother or older adults who do not know how to use technology and like the traditional way of interfacing with people. This opinion was shared by a Caucasian son (age 53) and an African American husband (age 66) who expressed apprehension about suitability of the online program for older caregivers. (p.172) | Unequivocal |
| Videos: helping caregivers understand provision of activities of daily living | Caregivers likewise expressed willingness to view videos focusing on the provision of assistance with activities of daily living throughout dementia stages. (p.170 ) | Credible |
| Videos: poor representation of more challenging situations | The Caucasian daughter (age 61) suggested the vignettes did not portray the “messiness of life”—times when a care recipient may not follow caregiver’s guidance, multiple family members involved in caregiving, and families with limited resources: I would have liked to see a daughter or son single caregiver with just a parent, try to make it more identifiable and inclusive . . .. (p.170)  several caregivers expressed the wish that the vignettes would portray more complex situations: The Jim and Becky [names of the fictional family] episodes were easy to identify with in most situations; however, Jim was an exceptionally agreeable individual and my husband still has an opinion of his own, which isn’t always the same as mine. I would have liked to see more tips on how to deal with resistance. (Caucasian wife, age 67) (p.170) | Unequivocal |
| Videos: Poor representation of cultural diversity | One African American niece (age 47) and one Caucasian daughter (age 61) noted lack of diversity in videos: only Caucasian actors and only a few non-Caucasian health care professionals appeared in them. (p.170)  One [caregiver] with many people meddling in the caretaking, I wish it had been not just Caucasian race, it’s not real life, nice suburban setting, my life doesn’t look like that with my mother. (p.170) | Unequivocal |
| Videos: promotes understanding | “To me the videos are key to the whole class” (member check participant). “Seeing the behavior depicted by the actors was a great way to better understand and grasp the different stages and how to adjust level of involvement” (Caucasian daughter, age 66). (p.170 ) | Unequivocal |
| Program designs: tailored to meet individual needs | “Much of the material was very basic for me, I would like more information on later stages because that’s where it really gets tough.” (p.168 )  “[I] may be younger, more dealing with mother-in-law and children; other people [were] older, a little disconnection in that way.” (p.169)  there were more strategies for early stages and handling awkward situations in early stages” (Caucasian wife, age 75) (p.169) | Unequivocal |
| Program design: lack of systematic layout of content and resources | Participants suggested that the manual be laid out more clearly (e.g., include a table of contents and a glossary) and be more precisely coordinated with the videos, videoconference “lectures,” and “homework” assignments. For many who attempted to complete all assignments, the lack of clear coordination was frustrating: “It skipped around all over the manual . . . it was a little confusing, the last thing you need is to be confused” (Caucasian wife, age 69). (p.171 ) | Unequivocal |
| Content design: relevant and targeted information | “It’s much better for those starting off . . . content gives a good picture of the first half of the journey. It does not relate at all to someone caring for a late-stage dementia” (Caucasian husband, age 72). (p.169)  “Too much! For me and my situation, it’s too hard. Make sure their situation is the right situation—the content wasn’t applicable to me yet.” (p.169)  “I was the only early stager. . . I am not experiencing what other people are experiencing. Put in more [information for] early stages” (Caucasian wife, age 60). (p.169) | Unequivocal |
| Program design: comprehensive instructions for navigation valued | Although instructions were provided, many caregivers recommended more practice and detailed written and illustrated instructions for video viewing (written instructions were not initially provided for video viewing). (p.169) | Credible |
| Program design: option for additional materials to share | Participants also asked for extra manuals to share with their family members and hard copies of PowerPoint slides presented during videoconferences. (p.171) | Credible |
| Program design: convenient for caregivers | “It was great being able to sit in the comfort of my home and interact with everyone. (Caucasian daughter, age 66). (p.168) | Unequivocal |
| Program design: flexibility of learning | Comments from a rural caregiver (Caucasian wife, age 76), “I live forty of miles from everywhere; it was wonderful,” and an urban caregiver (African American wife, age 73), “It was good to be able to do it online rather than trying to get in the car, considering the traffic situation here,” confirmed that the online format promoted access for those with travel challenges. (p.167) | Unequivocal |
| Accessibility: revisiting information when needed | Caregivers could not access the videos after Tele-Savvy conclusion; however, many stated that they would be willing to rewatch videos, share them with family members, and rewatch them when their care recipient is in a later dementia stage: “Your care recipient is not going through everything when it is discussed” (Caucasian husband, age 72). (p.171) | Unequivocal |

Lewis, M. L., Hobday, J. V., & Hepburn, K. W. (2010). Internet-Based Program for Dementia Caregivers. Am J Alzheimers Dis Other Demen, 25(8), 674-679. <https://doi.org/10.1177/1533317510385812>

| Findings | Illustration from the study | Evidence |
| --- | --- | --- |
| Peer support: valuing connection for sharing experience | One participant wrote, ‘‘I don’t have the option of sharing, or interacting with others. The opportunity for questions related to my situation are not possible (although I was amazed at how often the training content did relate to things we are dealing with even in the early stages).’’ (p.677) | Unequivocal |
| Hearing all perspectives empowers caregivers | ‘‘It is a gentle reference vehicle to understanding Alzheimer’s changes. It won’t smack you in the face with the fear of what is coming but will prepare you for techniques to cope.’’ | Unequivocal |
| Program designs: practical and relevant | ‘‘Good information, I found myself surprised at being able to relate to a lot of it.’’ Respondents identified the video examples as interesting. (p.677)  ‘‘Person with dementia was very interesting and I felt like I could connect with them.’’ (p.677) | Unequivocal |
| Variety and quality of presenters | ‘‘Good information attractively presented by a variety of speakers, both caregivers and experts.’’. (p.677) | Unequivocal |
| Program designs: Quality of grammar | Participants expressed concerns over spelling errors and navigation difficulties. | Credible |
| Content design: repetitive | ‘‘Same thing repeated over again in each module.’’ (p.677) | Unequivocal |
| Content designs: optimal length | ‘‘The length, I wished I could have watched it in one sitting.’’ (p.677) | Unequivocal |
| Content design: information overload | it was a lot of information to take in during one session. (p.678) | Unequivocal |
| Content designs: additional content required | additional content… such as information about medications and planning for the future decision making. (p.678) | Unequivocal |
| Program designs: unable to bookmark | ‘‘There was no way to mark where I left off each time, so I had to start over each time I returned to the program.’’ (p.677) | Unequivocal |
| Program designs: easier to navigate and revisit | making it easier to navigate and suggestions for specific edits to the program. (p.678) | Unequivocal |
| Program designs: hardcopy supplement | Accompany of a workbook for them to view in print. (p.678) | Credible |
| Program design: flexibility of learning | ‘‘I enjoyed having more control over when, where how long, and how much I worked.’’. (p.677) | Unequivocal |

Ploeg, J., McAiney, C., Duggleby, W., Chambers, T., Lam, A., Peacock, S., Fisher, K., Forbes, D. A., Ghosh, S., Markle-Reid, M., Triscott, J., & Williams, A. (2018). A web-based intervention to help caregivers of older adults with dementia and multiple chronic conditions: Qualitative study. JMIR Aging, 1(1), e2-e2. <https://doi.org/10.2196/aging.8475>

| Findings | Illustration from the study | Evidence |
| --- | --- | --- |
| Peer support: opportunity for caregivers to interact with each other | It’s nice seeing comments from other people, and there should be a lot more of that. Because you end up thinking—and I know it’s not the case—but you end up thinking that you’re the only one going through it, and then you realize that there’s an awful lot of other people doing it, too. [Participant 21, M3] (p.8) | Unequivocal |
| Peer support: peer confirmation of caregiving activities. | ...confirmation that you're not alone. [Participant 345, M1] (p.8)  It’s what’s to be expected, and not anything we didn’t do, or that we’re doing wrong, or, that we haven’t done. [Participant 390, M1] (p.8)  In each of the sections, I’ve well been there. I’ve been caregiving since 2008, and I have a large care manual that I started writing, and I’ve accumulated a lot of information...So you know, I have my objectives and I have long-term plans of certain things I’m going to do. So when I look at the website, I think I’m pretty well on track. [Participant 23, M1] (p.8) | Unequivocal |
| Peer support: valuing connection for sharing experience | Some participants commented that adding a feature to MT4C to enable caregivers to connect with one another to share information, experiences, and caregiving strategies would be helpful: ...if you connect with people over the internet say, you know, I’m having a really hard time today and somebody can say: “I know what you’re going through,” that can be good support too, you know? [Participant 399, M3] (p.9) | Unequivocal |
| Program encouraged caregiver to plan for future | ...look at what’s coming and plan for the future. [Participant 372, M3] (p.6)  ...But the long-term is what made me think...my husband and myself manage all her medical things, and it [MT4C] made me even realize somebody else needs a list of doctors and [chuckles] you know, things like that...It made me think about personal care in the future because that’s long-term care. [Participant 344, M1] (p.6)  Participants also talked about anticipating and planning for changes such as the care recipient’s move to assisted living or long-term care; arranging power of attorney; and anticipating changes to their living arrangements, such as making modifications to their existing home to accommodate the needs of the care recipient or moving to be closer to family and other forms of support. (p.6) | Unequivocal |
| Program facilitator: benefit of a link person /facilitator | having a person available to answer caregivers’ questions by telephone, and (3) having a navigator to “be that bridge” [Participant 373, M3] to help the caregiver identify and access resources that meet their specific needs.(p.9) | Unequivocal |
| Journal activities as part of program for reflection | ...you can type down something, type it in, and then it’s almost like a diary. And then kind of go back and go, “Hmm, I wonder, why did I put it that way? [Participant 322, M1] (p.5)  It [MT4C] allowed me to write down stuff that I haven’t stopped to write down, and I found that that was very helpful...just the opportunity to write down my story and how things have gone. It’s not something a caregiver takes time to do, and it’s really important...It makes you think of stuff that you sort of put in the back of your brain and it makes you put it down in front of you.[Participant 1, M1](p.5)  it made me take a deeper, inner look at myself, which I seldom do because I’m more focusing on [name of spouse] than I am on myself, and I’ve always found it a little difficult to focus on myself anyhow...it gave me a little chance of soul-searching and analyzing what I am doing and assessing some of the things I need to revise in my own thinking. So I found it very challenging and interesting. [Participant 301, M1](p.5)  ...the place where you had to make a list of the things that help you get through the day, [What Helps Me?] because I think it is such a negative situation and it’s so exhausting, physically and mentally and emotionally, that you could forget about that. So in the sense that it made you sit and think about it, I think that was a positive thing. [Participant 349, M3](p.5) | Unequivocal |
| Journal activities aid self-care | Well, the writing down of the stressful things that were happening; just the fact that I was able to share things and not keep it to myself, kind of thing. As I say, when all this happens, I’m on my own, and just the fact that I can share it is, you know, even if nobody reads it, the fact that I’ve took it out of my mind, there. So I did really find that helpful. [Participant 330, M3] (p.5)  I just found it more, therapeutic, I think, than anything else, to write down those things that I needed to think about. [Participant 383, M1] (p.5)  I did do some of the ones [sections of the website] of taking care of myself and...that’s one of my big things is that I understand that I really have to take care of myself, because I can’t help [name of spouse] if I’m not well. [Participant 337, M1] (p.5) | Unequivocal |
| Insufficient time for program participation | lack of time to use the site due to the demands of caregiving and other responsibilities. As one caregiver explained: The amount of time you had to sit and write things down, type things in, and to be honest with you, the more time I spend on the computer, the more [name of spouse] approaches me and saying “What are you doing? Why aren’t you sitting with me?” [Participant 353, M3] (p.9) | Unequivocal |
| Relevant content: applicable to caregivers’ situation | ...And even though I did do a lot of research, some of the stuff in there I hadn’t found before, so it helped me. [Participant 11, M1] (p.7)  I like the fact that you give out the telephone numbers and the contact information, national contacts, I think that’s great! That's information worth something to me. [Participant 365, M3] (p.8)  The Resources, that one meant more to me than anything else, that’s probably where I spent most of the time, the links to the legal stuff, I needed that because I wanted to find out about Powers of Attorney. [Participant 350, M3] (p.8) | Unequivocal |
| Relevant content: detailed dementia progression | And it [MT4C] gives you the information and very detailed description of each level of, where they’re at in their dementia process. I found that was much better than what other sites that I’ve read...so I was better able to reassess where I thought my husband was at, compared to other sites where I’ve used essentially the same sort of tool, but not worded in such a way that was really as helpful as it is on your site. [Participant 1, M1] (p.7) | Unequivocal |
| Program designs: need for locally relevant information | adding a directory of services that is searchable by postal code (p.9). | Unequivocal |
| Program designs: Very organised layout | Caregivers found the layout of MT4C to be “very well organized” [Participant 342, M3] (p.9) | Unequivocal |
| Program designs: consideration of literacy levels | Some caregivers suggested improvements to make MT4C more user-friendly. These included reducing the use of medical language and adjusting literacy levels and providing an overview of the content of the site. As one participant stated: ...it is a lot of text, and the literacy level. Oh, the other thing is it’s only in English...you need to make the language a bit simpler. [Participant 331, M3] (p.9) | Unequivocal |
| Content design: relevant and targeted information | For example, several participants indicated that they were already familiar with available resources and had already used or were using community-based services to answer their questions and address their caregiving concerns. (p.8)  ...didn’t really need it [MT4C] at this point. [Participant 379, M3] (p.8)  I feel like I’m not there yet; Mom’s still early, so some of the things are a bit more advanced, talking about getting help and that sort of thing. We aren’t at that stage yet so I could see maybe as things progress that maybe I’d be going back here to kind of have it as another resource. I think that’s probably the main thing, is I feel like I don’t need it yet. [Participant 345, M1] (p.8)  One caregiver who had been caring for her 89-year-old mother for almost 4 years expressed how she felt that the Toolkit could not help her because: I’ve figured out everything on my own. [Participant 35, M1] ...I’m at the end now. And for somebody new into the dementia journey, I think it’s a great tool...right now, because I’m emotionally wrecked, physically, financially it [MT4C] can’t help me now [laughs]. [Participant 35, M3] (p.9) | Unequivocal |
| Program design: flexibility of learning | ...something I could look at and use part of it or some of it, a little of it or none of it, but it gave me that basis to...sort of a mode of attack of how I was going to handle the situation. [Participant 301, M3] (p.7) | Unequivocal |
