# Supplementary material for: The Experiences of Informal Caregivers of People With Dementia in Web-Based Psychoeducation Programs: Systematic Review and Metasynthesis
Source: JMIR Aging. 2023 May 29;6:e47152. doi: 10.2196/47152 (PMC10262022; doi:10.2196/47152)
Supplement: Multimedia Appendix 5 [file aging_v6i1e47152_app5.docx]

**Appendix 5 Table Results of meta-synthesis**

| Findings | Category | Synthesised findings |
| --- | --- | --- |
| Program encouraged caregiver to plan for future (U) | Encouraging future planning | Synthesised finding 1:  Online learning as an empowering experience |
| Feeling of being supported by program providers (U) | Empowered through knowledge and support |  |
| Hearing all perspectives empowers caregivers (U) |  |  |
| Program facilitator: benefit of a link person /facilitator (U) | Facilitating accessing and utilising the program |  |
| Online class: like a real classroom (U) | Unexpectedly fun |  |
| Streamlined program provided positive learning experience (U) |  |  |
| Inspiring texts (U) | Inspiring |  |
| Journal activities as part of program for reflection (U) | A chance for reflection and self-care |  |
| Journal activities aid self-care (U) |  |  |
| Peer support: opportunity for caregivers to interact with each other (U) | Peer interactions | Synthesised Finding 2:  Peer support |
| Peer support: poor group interactions (U) |  |  |
| Peer support: Lack of equal opportunity to contribute (U) |  |  |
| Peer support: More interaction and discussions required (U) |  |  |
| Peer support: peer confirmation of caregiving activities (U) | Peer confirmation of caregiving activities. |  |
| Peer support: Reduced feelings of isolation (U) |  |  |
| Peer support: remaining in contact post-program (U) |  |  |
| Peer support: Preference for longer duration (U) | Peer connections |  |
| Peer support: valuing connection for sharing experience (U)(Ploeg et al., 2018) |  |  |
| Videos: related to disease progression (U) | Video content and display | Synthesised finding 3:  Satisfactory and unsatisfactory program contents |
| Videos: helping caregivers understand the provision of activities of daily living (C) |  |  |
| Video: relevant structure and content (C) |  |  |
| Videos: Poor representation of cultural diversity (U) |  |  |
| Videos: poor audio quality (C) |  |  |
| Videos: poor representation of more challenging situations (U) |  |  |
| Videos: poor visual display quality (U) |  |  |
| Videos: promotes understanding (U) |  |  |
| Relevant content: Information for the start of the caregiving journey (U) | Information applicability |  |
| Relevant content: accommodating caregivers learning needs (C) |  |  |
| Relevant content: applicable for other family members (C) |  |  |
| Relevant content: applicable to caregivers’ situation (U) |  |  |
| Program designs: practical and relevant (U) |  |  |
| Program designs: need for locally relevant information (U) |  |  |
| Program designs: tailored to meet individual needs (U) |  |  |
| Program design: lack of comprehensiveness (C) |  |  |
| Variety and quality of presenters (U) |  |  |
| Content design: relevant and targeted information (U) |  |  |
| Program designs: user friendly text (C) | Visual layout | Synthesised finding 4: Satisfactory and unsatisfactory technical design |
| Program designs: Very organised layout (U) |  |  |
| Program design: Learning unit structure (C) | Structure |  |
| Program design: lack of systematic layout of content and resources (U) |  |  |
| Program designs: Quality of grammar (C) | Language and literacy |  |
| Program designs: consideration of literacy levels (U) |  |  |
| Program design: negative case scenario (U) | Program content |  |
| Program design: Quizzes not suitable (C) |  |  |
| Content design: repetitive (U) |  |  |
| Content designs: optimal length (U) |  |  |
| Content design: information overload (U) |  |  |
| Content designs: additional content required (U) |  |  |
| Program designs: unable to bookmark (U) | functionality |  |
| Program designs: easier to navigate and revisit (C) |  |  |
| Program design: easy to navigate (U) |  |  |
| Program design: convenient for caregivers (U) |  |  |
| Program design: flexibility of learning (U) |  |  |
| Program design: comprehensive instructions for navigation valued (U) | supplementary material |  |
| Program design: option for additional materials to share (C) |  |  |
| Program designs: hardcopy supplement (C) |  |  |
| Accessibility: revisiting information when needed (U) | Accessibility |  |
| Accessibility: enhanced reach of program (U) |  |  |
| Difficulties with site access and navigation (U) | Difficulties in accessing online program | Synthesised findings 5:  Challenges encountered in online programs |
| Low level of computer literacy contributed to access difficulties (U) |  |  |
| Technical issues precluded engagement (C) | Reason for non-user |  |
| Too stressed to take part (U) |  |  |
| Insufficient time for program participation (C) |  |  |
| Personal preference for hard copy (U) | Personal preference for non-online program |  |
| Personal preference of actual social contact with others (U) |  |  |
