# Supplementary material for: The Experiences of Informal Caregivers of People With Dementia in Web-Based Psychoeducation Programs: Systematic Review and Metasynthesis
Source: JMIR Aging. 2023 May 29;6:e47152. doi: 10.2196/47152 (PMC10262022; doi:10.2196/47152)
Supplement: Multimedia Appendix 6 [file aging_v6i1e47152_app6.docx]

**Appendix 6 The ConQual summary of findings**

| **Systematic review title:** The experience of informal caregivers of people with dementia in online psychoeducation programs: a systematic review and meta-synthesis of qualitative studies  **Population:** Informal caregivers of people with dementia  **Phenomena of interest:** Online psychoeducation programs | | | | |
| --- | --- | --- | --- | --- |
| Synthesised findings | Type of research | Dependability | Credibility | ConQual score |
| **Synthesised Finding 1: Online learning as an empowering experience** | Qualitative and qualitative components in mixed-method research | High | High | High |
| **Synthesised Finding 2: Peer support** | Qualitative and qualitative components in mixed-method research | High | High | High |
| **Synthesised Finding 3: Satisfactory and unsatisfactory program content** | Qualitative and qualitative components in mixed-method research | High | Downgrade 1 level | Moderate |
| **Synthesis finding 4: Satisfactory and unsatisfactory technical design** | Qualitative and qualitative components in mixed-method research | High | Downgrade 1 level | Moderate |
| **Synthesised Finding 5: Challenges encountered in online learning** | Qualitative and qualitative components in mixed-method research | High | High | High |

Note: Dependability: high= 4-5 responses to critical appraisal questions. Credibility: high=unequivocal: all findings accompanied by an illustration (no change); downgrade one level due to a mix of unequivocal and credible findings.
