# Supplementary material for: The Experiences of Informal Caregivers of People With Dementia in Web-Based Psychoeducation Programs: Systematic Review and Metasynthesis
Source: JMIR Aging. 2023 May 29;6:e47152. doi: 10.2196/47152 (PMC10262022; doi:10.2196/47152)
Supplement: Multimedia Appendix 7 [file aging_v6i1e47152_app7.docx]

**Appendix 7 The meta-aggregation flow chart**

Identification

Findings

Categorisation

Synthesis

Review questions: is what are informal caregivers’ experiences in online psychoeducation programs

Search for appropriate studies

(Refer to PRISMA flow chart)

Critical appraisal and selection of studies

**Study methodologies**

Qualitative descriptive, content analysis, case study

Aggregate 87 unequivocal and credible findings from 9 studies into 20 categories

Synthesise 20 categories into 5 synthesised findings

**Five synthesised findings**

1. Online learning as an empowering experience

2. Peer support

3. Satisfactory and unsatisfactory program content

4. Satisfactory and unsatisfactory technical design

5. Challenges encountered in online learning

**20 categories**

1. Encouraging future planning

2. Empowered through knowledge and support

3. Facilitating accessing and utilising the program

4. Unexpected fun

5. Inspiring

6. A chance for reflection and self-care

7. Peer interactions

8. Peer confirmation of caregiving activities

9. Peer connections

10. Video content and display

11. Information applicability

12. Visual layout

13. Structure

14. Program content

15. Functionality

16. Supplementary material

17. Accessibility

18. Difficulties in accessing online programs

19. Reason for non-user

20. Personal preference for the non-online program

**Recommendations**

1. Offering tailored information to meet individual learning needs

2. Programs should facilitate social

connections among caregivers

3. Having health and social care professionals as program facilitators to provide individualised support

4. Integrating multi-modality teaching and learning materials in one program

5. Offering asynchronised online teaching and learning to accommodate a broader audience, especially working caregivers

6. Program development should base on needs analysis. The provider should conduct an ongoing evaluation of the program

7. Offer initial training and technical support

8. Future qualitative research that explores caregiver's experiences are needed
